# Fibromodulin reduces scar formation in adult cutaneous wounds by eliciting a fetal-like phenotype

**DOI:** 10.1038/sigtrans.2017.50

**Published:** 2017-10-13

**Authors:** Zhong Zheng, Aaron W James, Chenshuang Li, Wenlu Jiang, Joyce Z Wang, Grace X Chang, Kevin S Lee, Feng Chen, Emily A Berthiaume, Yao Chen, Hsin Chuan Pan, Eric C Chen, Weiming Li, Zhihe Zhao, Xinli Zhang, Kang Ting, Chia Soo

**Affiliations:** 1 Division of Growth and Development, School of Dentistry, University of California, Los Angeles, Los Angeles, CA 90095, USA; 2 UCLA Division of Plastic and Reconstructive Surgery, Department of Orthopaedic Surgery, The Orthopaedic Hospital Research Center, University of California, Los Angeles, Los Angeles, CA 90095, USA; 3 Department of Pathology, Johns Hopkins University, Baltimore, MD 21287, USA; 4 State Key Laboratory of Oral Diseases, Department of Orthodontics, West China School of Stomatology, Sichuan University, Chengdu, Sichuan 610041, China; 5 Department of Emergency Medicine, Highland General Hospital, Oakland, CA 94602, USA; 6 David Geffen School of Medicine, University of California, Los Angeles, Los Angeles, CA 90095, USA; 7 Central Laboratory, School of Stomatology, Peking University, Beijing 100081, China; 8 Department of Orthopaedics, the First Clinical Hospital of Harbin Medical University, Harbin, Heilongjiang 150081, China

## Abstract

Blocking transforming growth factor (TGF)β1 signal transduction has been a central strategy for scar reduction; however, this approach appears to be minimally effective. Here, we show that fibromodulin (FMOD), a 59-kD small leucine-rich proteoglycan critical for normal collagen fibrillogenesis, significantly reduces scar formation while simultaneously increasing scar strength in both adult rodent models and porcine wounds, which simulate human cutaneous scar repair. Mechanistically, FMOD uncouples pro-migration/contraction cellular signals from pro-fibrotic signaling by selectively enhancing SMAD3-mediated signal transduction, while reducing AP-1-mediated TGFβ1 auto-induction and fibrotic extracellular matrix accumulation. Consequently, FMOD accelerates TGFβ1-responsive adult fibroblast migration, myofibroblast conversion, and function. Furthermore, our findings strongly indicate that, by delicately orchestrating TGFβ1 activities rather than indiscriminately blocking TGFβ1, FMOD elicits fetal-like cellular and molecular phenotypes in adult dermal fibroblasts *in vitro* and adult cutaneous wounds *in vivo*, which is a unique response of living system undescribed previously. Taken together, this study illuminates the signal modulating activities of FMOD beyond its structural support functions, and highlights the potential for FMOD-based therapies to be used in cutaneous wound repair.

## Introduction

Cutaneous wounds, acquired from surgery or trauma, can cause pathologic scarring with significant functional and psychological sequelae. These pathologies cost the global healthcare system over $8.6 billion and affect 100 million patients annually.^1,2^ Unfortunately, knowledge of processes regulating dermal wound healing is still incomplete, which leaves patients with suboptimal treatment options. For example, the current standards of care for cutaneous scarring exhibit numerous undesirable side effects:^3^ Local corticosteroid injections can result in reduced scar strength, pigmentation changes, granulomas, and skin atrophy, while wound irradiation can cause growth inhibition, decreased scar strength, and increased long-term cancer risks.

Although multiple growth factors and cytokines contribute to cutaneous repair, transforming growth factor (TGF)β1 has been recognized as a central modulator of wound healing.^4,5^ The well-documented inflammatory and pro-fibrotic effects of TGFβ1 have precipitated a considerable body of research and clinical efforts in TGFβ1 signaling blockade to reduce scarring (‘low TGFβ1’ strategies).^4,6^ However, application of TGFβ1-neutralizing antibodies did not effectively prevent scar formation in adult rat cutaneous wounds.^7^ Additionally, a synthetic inhibitor peptide of TGFβ failed to reduce fibrosis in a porcine silicone pericapsular fibrotic model.^8^ Beyond this, in human clinical trials, the knock-down of connective growth factor (CTGF) expression, a known downstream mediator of TGFβ1 bioactivity,^9^ was inadequate in reducing scar formation.^10,11^ Even though studies have examined TGFβ1 signaling blockades for possible benefits, it is important to consider off target effects of TGBβ1 blockade. Transgenic mice studies reveal that simple TGFβ1 blockade does not promote ideal wound healing, and instead, TGFβ1-deficient mice die from overwhelming systemic inflammation within 3–4 weeks after their birth.^12^ When crossed with severe combined immunodeficiency mice, TGFβ1-deficient mice survive but exhibit impaired granulation tissue formation and delayed cutaneous wound repair.^13^ Finally, inducible fibroblast lineage-specific type II TGFβ receptor-knockout mice demonstrate severely impaired fibroblast migration, myofibroblast development, and myofibroblast function, which leads to deficient granulation tissue formation and defective wound contraction.^14^ These results indicate that TGFβ1 signaling has multiple roles in the wound healing process, and as such, non-specific TGFβ-blockade is detrimental to cutaneous repair.

Before a particular gestation age, fetal cutaneous wounds can heal scarlessly with apparent restoration of the normal dermal extracellular matrix (ECM) architecture and dermal appendages.^15^ In rodents, skin wounds begin to scar at approximately day 18.5 of a roughly 20-day gestation period, whereas in humans, cutaneous scarring begins approximately at week 24 of gestation.^15^ Accumulating data indicate that fetal-type scarless healing is likely inherent to the unique properties of fetal cells, ECM, and growth factor/cytokine profiles that comprise fetal skin, rather than the intrauterine environment.^15^ Using a rodent fetal wound healing model, we previously reported a rapid decline in fibromodulin (FMOD) expression coinciding with the transition from fetal-type scarless repair to adult-type scarring repair.^16,17^ FMOD is a 59-kD small leucine-rich proteoglycan (SLRP) with well-described roles in collagen fibrillogenesis.^18^ FMOD loss- and gain-of-function models have assisted in confirming the importance of FMOD in fetal-type scarless repair, as well as determining TGFβ ligand and receptor levels in fetal and adult skin.^16,17^ In addition, FMOD deficiency in adult mice has been shown to increase scar size and elicit a severely deficient dermal fibroplasia response, which is characterized by profoundly impaired fibroblast migration and delays in granulation tissue formation.^19^ Both of these attributes are strikingly similar to those of the inducible, fibroblast lineage-specific TβRII-knockout mice.^14^

Collectively, these results suggest that both FMOD and TGFβ signal transduction are critical to early dermal fibroblast function during fetal and adult wound repair. We previously described that FMOD deficiency markedly alters the spatiotemporal expression patterns and levels of TGFβ ligands and receptors during adult mouse wound healing.^19,20^ These findings indicate that FMOD is required for proper TGFβ expression and function during wound repair. In this report, we systematically investigated the interplay between FMOD and TGFβ1 for their roles in the regulation of fibroplasia during adult cutaneous wound repair in preclinical animal models among different species, including mouse, rat and pig.

## Materials and methods

### Study design

The objective of this study was to assess the effects of FMOD on adult dermal fibroblasts and its therapeutic potential for adult cutaneous wound healing. For all *in vitro* studies, we used adult rat dermal fibroblasts (RDFs) since dermal fibroblasts are the predominant cell type required for cutaneous wound repair. Primary closure wound models were used in this study to simulate post-surgical wounds, which occur in 55 million elective operations and 25 million traumatic injury operations annually.^2^ Management of the resulting unwanted scarring requires approximately $3 billion each year.^2^ To begin, we used mouse and rat cutaneous wounds to test the efficacy of FMOD. Rodent animals were randomly assigned to each experimental group, and the sample size was determined based on previous studies.^19–21^ Rodents are loose-skinned animals, and as such, their skin can slide and retract over the subcutaneous fascia to produce a large gap initially.^22^ On the contrary, the pig and human skin is firmly attached to the underlying structure.^23,24^ Accordingly, a porcine model was then chosen for clinical relevance.^23,24^ Porcine wounds were randomly treated with phosphate-buffered saline (PBS) control or FMOD among different pigs. Initial porcine wound numbers were determined using power analysis to give *α*=0.05 and power=0.8, based on preliminary data. All animal surgeries were performed in accordance with the NIH Guide for the Care and Use of Laboratory Animals set forth under the institutionally approved protocols provided by the Chancellor’s Animal Research Committee at UCLA (protocol number: 2000-058 and 2008-016). Sutures were removed after stable wound closure based on our preliminary observations (1- (for rodent) or 2 weeks (for pig) post-injury). Early termination of animal experiments was based on either significant morbidity for animals or a loss of body weight more than 10%, in accordance with the approved protocols provided by the Chancellor’s Animal Research Committee at UCLA, although no animals were sacrificed for early termination during this study. The number of experimental replicates is defined in each figure legend. All outliers were included in the analysis, and no data were excluded. Investigators were blinded to treatment groups in all subjective *in vivo* measurements.

### FMOD production

cDNA of a human FMOD transcript (Genbank assessor number: NM_002023) was subcloned into a commercially available vector pSecTag2A (Life Technology, Grand Island, NY, USA) with C-terminal His-tag, and transfected into CHO-K1 cells (ATCC, Manassas, VA, USA).^19^ After establishing a stable expression clone, the FMOD was produced and purified by a contract research organization, GenScript (Piscataway, NJ, USA). Briefly, a stable human recombinant FMOD-expressing CHO-K1 cell line was cultured in 1 l serum-free Freestyle CHO Expression Medium (Thermo Fisher Scientific, Canoga Park, CA, USA) at 37 °C with 5% CO_2_ in an Erlenmeyer flask. Cell culture supernatant was harvested on day 10 for purification with HiTrap IMAC HP, 1-ml column (GE Healthcare, Uppsala, Sweden). The fractions from a 100 mm imidazole elution were collected and dialyzed against 20 mm PBS, pH 7.4. After that, the sample with low conductivity was loaded onto HiTrapQ HP 1-ml column (GE Healthcare) for further purification. FMOD was then purified under non-reducing conditions, dialyzed again,^25^ and then subjected to lyophilization. The purity of the FMOD product is 85%. FMOD is reconstituted in PBS, followed by sterilization through a 0.22-μm filter (Thermo Fisher Scientific) before usage.

### Adult rat skin wound model

Adult male Sprague-Dawley (SD) rats (weighing ~300 g) were anesthetized, and the dorsal skin was sterilely prepared. Six full-thickness, 10 mm×3 mm skin ellipses, with the underlying panniculus carnosus muscles, were excised on the dorsum of each animal. Each open wound edge was injected with 25 μl PBS, or 25 μl 0.4 or 2.0 mg ml^−1^ FMOD in PBS (25 μl×2 edges=50 μl total/wound). For the inhibitor-FMOD combination treatment groups, SMAD3-specific inhibitors (described below) were used with 2.0 mg ml^−1^ FMOD. Wounds were then marked with permanent dye and closed primarily with 4-0 Nylon using two simple interrupted sutures consistently placed at one-third intervals in each 10-mm length wound. All wounds were separated by at least 2 cm to minimize adjacent wound effects. Sutures were removed 1 week after injury, and wounds were collected 2 weeks after injury. Skin tissues from identical locations of unwounded animals were collected as controls. Wounds were harvested by excising a 4 mm×2 mm full-thickness skin strip, which was divided in two along its short axis.

### Adult mouse skin wound model

Three-month old male 129/sv wild-type (WT) and *Fmod-null* (*Fmod*^*−/−*^) mice were anesthetized, and the dorsal skin was sterilely prepared. Four full-thickness, 10 mm×3 mm skin ellipses, with the underlying *panniculus carnosus* muscles, were excised on each mouse. Each open wound edge was injected with 25 μl PBS, 25 μl 0.4 mg ml^−1^ FMOD in PBS, or left untreated (25 μl×2 edges=50 μl total per wound). Wounds were then primarily closed with 5-0 Nylon using two simple interrupted sutures consistently placed at one-third intervals in each 10-mm length wound. All wounds were separated by at least 2 cm to minimize adjacent wound effects. Sutures were removed day 7 post-injury, and wounds were harvested 14 days post-injury (9 separate animals for each genotype; *N*=18 wounds per condition).^19,26^

### Porcine wound healing model

Primary closure wound porcine models were used in this study, as described previously.^24^ Briefly, 20-kg female Yorkshire pigs (S&S Farms, Ramona, CA, USA) were sedated with Telazol (Tiletamine and Zolazepam, Fort Dodge Animal Heath, Fort Dodge, IA, USA), a 5 mg kg^−1^ intramuscular injection. The pigs were then endotracheally intubated, and maintained under a surgical plane of anesthesia with isoflurane at 0.5–2.5% in room air. The flank and back hair was clipped, and the skin was sterilely prepared with three alternating scrubs of povidone iodine solution and alcohol. Since porcine skin is significantly thinker than rodent skin, full-thickness wounds, down to the fascia, were created with a #15 surgical blade by excising a 1.5 cm×0.5 cm ellipse of skin with its long axis running perpendicular to the lines of minimal tension. All wounds were separated by at least 2 cm to minimize adjacent wound effects. Each open wound edge was injected with 100 μl PBS or 2.0 mg ml^−1^ FMOD in PBS (100 μl×2 edges=200 μl total/wound). Wounds were then marked with permanent dye, and primarily closed with 3-0 Nylon mattress sutures. 24 h later, an additional 50 μl PBS or FMOD was injected into each edge of the wound. A total of 24 wounds were created in each animal. Sutures were removed 2 weeks post-injury, and wounds were harvested at either 4 or 8 weeks post-injury (*N*=6 wounds from 3 Yorkshire pigs). Unwounded skin tissues, distant from the wounds of each animal, were collected as control samples. Tissues were bisected centrally between the sutures and perpendicular to the long axis of each wound.

### Visual appearance evaluation of scarring

A gross visual assessment of the scar was performed in a randomized, double-blind fashion with an adaptation of Visual Analogue Score (VAS), as described previously.^27^ Briefly, all scar images were taken by a highly sensitive digital camera (DSLR DS126181, Canon, Tokyo, Japan) and individually presented to 3 experienced medical doctor (MD) assessors for a maximum of 20 s. The assessors placed a mark on a horizontal, 100-mm line to represent the scar quality, with 0 indicating unwounded skin and 100 indicating a poor scar (which represents a raised, hyperpigmented, or red scar with less acceptability and observer comfort; Supplementary Figure 1). When the individual images were displayed, the assessors confirmed and documented a VAS score independently.^27^

### Tensile strength measurement

Tensile strength was determined using an Instron 5565 Universal Testing Machine (Instron, High Wycombe, UK). Using pneumatic grips to avoid specimen slippage, an exact 4 cm×1 cm full-thickness rat or pig skin strip was obtained by meticulous dissection, with the sample being obtained by precisely bisecting the wound. A 1-cm square area of skin was clamped on either side of the wound. The load to failure (breaking strength), measured in Newtons (N), was recorded.

### Histological and immunohistological staining

After fixation in 10% neutral buffered formalin (Thermo Fisher Scientific) for 24 h, skin samples were dehydrated, paraffin-embedded, and sectioned at 5-μm increments for either hematoxylin and eosin (H&E) or Masson’s trichrome staining, and 10-μm increments for Picrosirius red (PSR) staining. To ensure a more precise quantification, wounds were bisected centrally, and the total scar area was normalized to varying dermal thickness using the Scar Index, as previously described.^19,26^ Among these staining methods, PSR coupled polarized light microscopy (PLM) distinguishes clear boundaries between scar tissue and unwounded normal dermis (Supplementary Figure 2). Immunohistochemical staining was performed and analyzed, as previously described.^19,20^ The primary antibodies used are listed in Supplementary Table 1.

### Confocal laser scanning microscopy

Following PSR staining, the dermal collagen deposition pattern of the upper dermis was evaluated by confocal microscopy on a Carl Zeiss LSM 510 META laser scanning confocal microscope (Carl Zeiss AG, Oberkochen, Germany), using previously published methods.^17,19,26^ Collagen organization architecture was assessed by Fractal dimension (*F*_*D*_) and Lacunarity (*L*) analyses, as previous described,^26^ since *F*_*D*_ and *L* analyses are more sensitive than traditional methods, such as polarized light microscopy (PLM), X-ray diffraction, laser scattering, and Fourier transform analysis.^26^

### RT^2^ profiler PCR array analysis of rat wounds

To minimize the contamination of the surrounding unwounded tissue, wound tissues were collected for RNA isolation by manual microdissection from paraffin-embedded tissue sections.^28^ Total RNA was isolated using RNeasy FFPE Kit (Qiagen, Hilden, Germany). 2.5 μg RNA isolated from the wounds was injected into RT^2^ First Stand Kit (Qiagen) for reverse transcription. Afterward, real-time PCR was performed in a 96-well rat wound healing RT^2^ PCR Array (PARN-121A, Qiagen) on a 7300 Real-Time PCR system (Thermo Fisher Scientific), according to the manufacturer’s protocol. For each sample, three arrays were tested. Data analysis was achieved using the manufacturer’s online services.

### RDF isolation and maintenance

Adult RDFs were isolated from the dorsal skin of adult male SD rats and maintained in Dulbecco’s modified Eagle’s medium (DMEM; Thermo Fisher Scientific) supplemented with penicillin/streptomycin (1% v/v; Thermo Fisher Scientific) and fetal bovine serum (10% v/v; Thermo Fisher Scientific) as previously described.^29^ RDFs were tested negatively for mycoplasma contamination by the Universal Mycoplasma Detection Kit (ATCC, Manassas, VA, USA; Supplementary Figure 3). RDFs at passage 3 were used for all *in vitro* tests.

### Cell proliferation assay

RDFs were seeded at a density of 2×10^3^ cells per well on 96-well cell culture plates. Since serum is a known source of multiple growth factors, including TGFβ1, serum starvation was carried out to standardize the cell cycle phase and eliminate the influence of growth factor persistence to accurately assess the bioactivities of FMOD. After 16-h serum starvation, cells were treated with 100 μl fresh medium containing FMOD and/or TGFβ1 (Sigma-Aldrich, St Louis, MO, USA). After 48-h incubation, cell proliferation was measured by the Vybrant MTT Cell Proliferation Assay Kit (Thermo Fisher Scientific).

### Cell migration assay

For wound scratch assays, RDFs were grown in 6-well tissue culture plates until confluence. After 16-h serum starvation, 1-mm width ‘scratch’ wounds were created by scraping confluent cell monolayers using a sterile pipette. To accurately assess cellular migration, each scratch was examined immediately after scraping under microscope. Only the scratches with a width of 1±0.1 mm were used for further investigation. The wounded monolayer was washed three times with PBS to remove dead cells prior to 20-h incubation in treatment medium: DMEM±2.5 ng ml^−1^ (100 pm) TGFβ1±12 μg ml^−1^ (200 nm) FMOD. Concentrations of TGFβ1 and FMOD used were based on previous report.^19^ Photographs taken immediately after scraping and 20 h later documented cell migration. Migration was quantified by measuring the average wound gaps between the wound edges before and after treatment, using the commercially available software Image-Pro Plus 6.0 (Media Cybernetics, Rockville, MD, USA).

### Cell invasion assay

Cell invasion assays were performed in 24-well tissue culture plates using HTS Fluoroblok inserts with 8 μm pore size Fluorescence Blocking PET track-etched membranes (BD Biosciences, Franklin Lakes, NJ, USA). The upper surface of the inserts were coated with 200 μl collagen matrices, rinsed with DMEM, and placed into 24-well tissue culture plates containing 500 μl treatment medium (as described above). After 16-h serum starvation, 2×10^4^ RDFs in 100 μl treatment medium were added to each insert chamber and allowed to invade toward the underside of the membrane for 20 h. Non-invading cells were removed by wiping the upper side of the membrane with a cotton swab. Cells that invaded were fixed and stained with 0.4 mg ml^−1^ 4′,6-diamino-2-phenlindole (DAPI; Sigma-Aldrich) before counting.^19^

### Immunocytochemical staining

RDFs were seeded at a density of 1×10^4^ cells per well on 4-well Lab-TEK II chamber slides (Thermo Fisher Scientific). After 16 h serum starvation, cells were incubated with treatment medium (as described above) for 48 h before immunostaining. Images were obtained using a Leica TCS-SP2-AOBS confocal microscope (Leica Microsystems, Buffalo Grove, IL, USA). The primary antibodies used are listed in Supplementary Table 1.

### Flow cytometry

RDFs were harvested 48 h after treatment and filtered through a 40-μm strainer. Cell counts and viability were determined using trypan blue exclusion (Sigma-Aldrich) on a hemocytometer. Single cell suspensions were fixed with 4% formaldehyde, permeabilized with 0.1% Triton X-100 for 15 min, and then blocked with 3% BSA for 30 min before being incubated with α-smooth muscle actin (α-SMA) antibody (Abcam, Cambridge, UK) for 30 min at room temperature. Samples were assayed immediately by LSRFortessa Cytometer (BD Biosciences), and FCS Express 4 software (De Novo Software, Glendale, CA, USA).

### Collagen-based cell contraction assay

After 16-h serum starvation, 2×10^6^ RDFs per well were used to establish a 3D collagen matrix in a 24-well plate following the manufacturer’s instructions (Cell Contraction Assay, Cell Biolabs, San Diego, CA, USA). After collagen polymerization, 1.0 ml of treatment medium (as described above) was added atop each collagen gel lattice. Cultures were incubated for 2 days for stress development. Afterwards, collagen gels were gently released from the sides of the culture dishes with a sterile spatula. The collagen gel size changes were measured at 0, 20, 40 and 60 min.

### Western blotting

RDFs were seeded at a density of 1×10^5^ cells per dish on 10-cm tissue culture dishes. After 16 h serum starvation, cells were incubated with treatment medium (as described above). Cells were lysed using RIPA buffer (Thermo Fisher Scientific) supplied with Halt Protease and Phosphates Inhibitor Cocktail (Thermo Fisher Scientific) for Western blotting. The antibodies used in this study are also summarized in Supplementary Table 1. Bands on Western blots were quantified using QuantityOne (BioRad Laboratories, Hercules, CA, USA), and values were expressed in relative densitometry units.

### Cellular gene expression assay

RDFs were seeded at a density of 1×10^5^ cells per dish on 10-cm tissue culture dishes. After 16 h serum starvation, cells were incubated with treatment medium (as described above). RNAs were extracted using RNeasy Mini Kit (Qiagen) with DNase (Qiagen) treatment, followed by reverse transcription with SuperScript III First-Strand Synthesis System for RT-PCR (Thermo Fisher Scientific). qRT-PCR was performed on a 7300 Real-Time PCR system (Thermo Fisher Scientific) according to the manufacturer’s protocol.^30^ The primers and probes used in this study are listed in Supplementary Table 2. For each assay, at least three separate sets of qRT-PCR were performed from a different cDNA template. Concomitant glyceraldehyde-3-phosphate dehydrogenase (*Gapdh*) was performed in separate tubes, as a house-keeping standard since the _Δ_C_T_ levels of *Gapdh* did not differ significantly among the treatment conditions (data not shown). Relative gene expression was analyzed with the _Δ_C_T_ method.^19,20,30^

### Luciferase assays

RDFs were seeded at a density of 1×10^5^ cells per dish on 10-cm tissue culture dishes. After 16 h serum starvation, cells were transiently transfected with the A3-Luc system (that is, CAGA-box reporter; specific for SMAD3 signal transduction)^31^ or the Cignal AP-1 Reporter (luc) Kit (Qiagen; for AP-1 signal transduction). Luciferase activity was quantified at 2 and 24 h using the Dual Luciferase Assay (Promega, Madison, WI, USA). Values were normalized by Renilla luciferase activity expressed from pRL-TK (Promega).

### ELISA

ELISA of matrix metalloproteinase (MMP)2 was performed using the commercially available MMP-2 Quantikine ELISA Assay Kit (R&D Systems, Minneapolis, MN, USA). Briefly, 50 μl of provided diluent solution and 50 μl of conditioned cell culture supernatant were added to each well, and incubated at room temperature for 2 h. Following incubation, the wells were washed four times with 400 μl of provided wash buffer. Subsequently, 200 μl of MMP2 conjugate was added to each well and incubated at room temperature for 2 h before being washed with 200 μl of provided wash buffer four times, and incubated with provided 200 μl of substrate solution at room temperature for 30 min. Afterward, 50 μl of stop solution was added to each well. MMP2 activity was measured using microplate reader (BioTek Instruments, Winooski, VT, USA) at 450 nm, subtracted by readings at 540 nm. Mmp2 concentrations were then calculated by creating a standard curve. All experiments were performed in triplicate.

### Inhibitors

SMAD3-specific inhibitors naringenin (4′,5,7-trihydroxyflavanone, which selectively inhibits the expression of SMAD3, but not SMAD2, SMAD4 or SMAD7;^32^ Sigma-Aldrich; 50 μM) and SIS3 ((2E)-1-(6,7-Dimethoxy-3,4-dihydro-1H-isoquinolin-2-yl)-3-(1-methyl-2-phenyl-1H-pyrrolo(2,3–b]pyridin-3-yl)-propenone hydrochloride, CAS 1009104-85-1, which attenuates phosphorylation of SMAD3, but not SMAD2, MAPK, ERK, or PI3-K;^33^ Calbiochem, San Diego, CA, USA; 5 μM) were used to block SMAD3 signal transduction.

### Statistical analysis

All statistical analyses were conducted in consultation with the UCLA Statistical Biomathematical Consulting Clinic. Statistical analysis was computed by OriginPro 8 (Originlab, Northampton, MA, USA). Data were generally presented as mean±s.d., and compared by one-way analysis of variance (ANOVA) and two-sample *t-*tests. Mann–Whitney and Kruskal–Wallis ANOVA tests were used for non-parametric data. *P*<0.05 was considered statistical significance.

### Data availability

All data generated or analyzed during this study are included in this article (and its Supplementary Information files).

## Results

### FMOD significantly optimizes adult cutaneous wound healing

**Figure 1:**
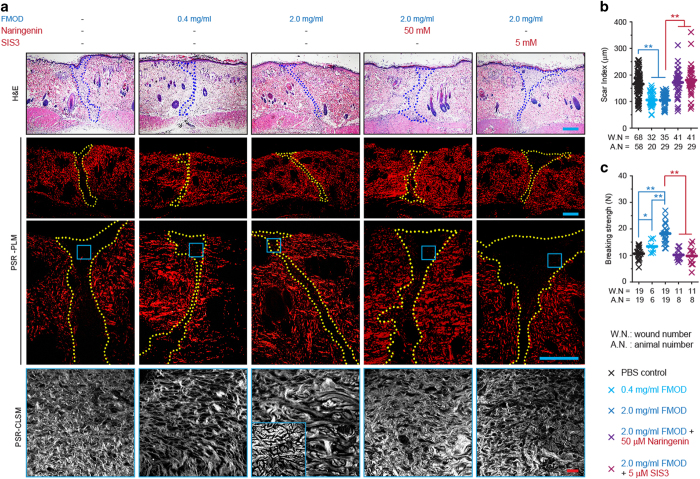
FMOD reduced scar formation and increased wound tensile strength in adult rat cutaneous wounds. Histology (H&E staining), picrosirius red (PSR) staining-coupled polarized light microscopy (PLM), PSR staining-coupled confocal laser scanning microscopy (CLSM) (**a**; insert, unwounded skin), and quantitative analyses (**b**) demonstrated that FMOD significantly reduced the scar size of adult rat skin wounds at day 14 post-injury, while increasing tensile strength in a dose-dependent manner (**c**). Moreover, SMAD3-specfic inhibitors naringenin and SIS3 eliminated the effects of FMOD on reducing scar formation and increasing wound tensile strength. Scar areas are outlined by blue (H&E) or yellow (PSR-PLM) lines. Scale bar=500 μm (blue) or 25 μm (red). Mann–Whitney test was used for statistical analysis, while mean values are presented. Wound numbers (WN) and animal numbers (AN) are indicated in the figure. *P<0.05, **P<0.01.

Previously, we reported that wounds in the adult *Fmod*^*−/−*^ mouse healed with increased scar formation, delayed wound closure, and reduced angiogenesis, which could be partially rescued by exogenous FMOD administration.^19,21^ Additionally, FMOD administration markedly augmented cutaneous wound vascularity^21^ and notably decreased scar size in adult WT mice (Supplementary Figure 4). Using an adult rat wound healing model, we demonstrated that exogenous FMOD significantly reduced scar size when compared with PBS control samples as evidenced by PSR staining-coupled PLM (Figure 1a and b). Meanwhile, FMOD-treated adult rat wounds also demonstrated and orderly collagen architecture as documented by PSR staining-coupled confocal laser scanning microscopy (CLSM) (Figure 1a), which was accompanied by increased tensile strength (Figure 1c).

**Figure 2:**
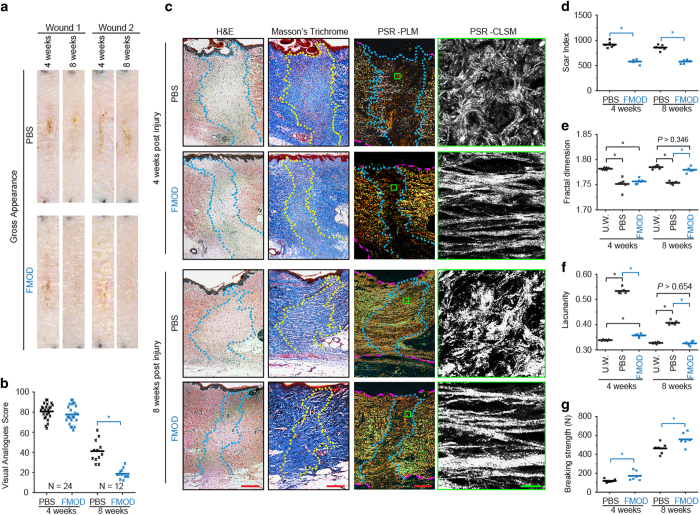
FMOD reduced scar formation and increased wound tensile strength in adult Yorkshire pig cutaneous wounds. Representative photographs of the gross appearance of adult female Yorkshire pig wounds at 4 and 8 weeks post-injury are shown (**a**). Compared with PBS-treated wounds, 2.0 mg ml^−1^ FMOD-treated wounds exhibited improved gross appearance (as evidenced by a decreased Visual Analogue Score; **b**). Histology of adult female Yorkshire pig wounds at 4 and 8 weeks post-injury are shown (**c**). Scars are outlined by blue or yellow lines, while dermis layers are outlined by dashed magenta lines in PSR-PLM photographs. Quantitative analyses demonstrated that FMOD significantly reduced scar size (scar index; **d**). Collagen architecture was evaluated by Fractal dimension (**e**) and Lacunarity (**f**) analyses. FMOD treatment also significantly increased wound tensile strength in comparison with PBS control (**g**). UW, unwounded skin control. Scale bar=500 μm (red), or 25 μm (green). Mann–Whitney test was used for statistical analysis, while mean values are presented. *N*=24 (**b**, 4 weeks post-injury), 12 (**b**, 8 weeks post-injury), or 6 (**d-g**) wounds from 3 pigs per time point. *P<0.05.

Among mammalian skin, porcine skin most closely approximates human skin in anatomic structure, mechanical properties, and wound healing dynamics.^23,24^ In order to rigorously test the wound healing effects of FMOD treatment in a model that more closely approximates human skin, we excised and primarily closed skin wounds in adult female Yorkshire pigs. FMOD injection at the time of wound closure significantly improved the gross appearance of the pig wounds when assessed at 8 weeks post-injury (Figure 2a and b). Using PSR-PLM, which distinguishes clear boundaries between scar tissue and unwounded dermis, FMOD-treated Yorkshire pig wounds exhibited significantly reduced scar size compared with controls when measured at 4 and 8 weeks post-injury (Figure 2c and d). The dermal collagen architecture was also assessed by PSR-CLSM (Figure 2c), and then quantified *F*_*D*_ and *L* analyses.^26^ *F*_*D*_ provides a measure of how completely an object fills space, which quantifies an object by shape, regularity, lack of smoothness, size, and number of self-similarities, that is, invariance regardless of scale.^34^ In general, a higher *F*_*D*_ value indicates a uniform distribution.^34^ For example, wounds have lower mean *F*_*D*_ values when compared with unwounded skin, corresponding to the uneven and disorganized collagen distribution patterns (Figure 2e). In contrast, *L* permits an analysis of density, packing, or dispersion through scales.^34^ Objects with lower *L* values correspond to a finer texture, while objects with higher *L* values are more spatially unorganized.^35^ In this study, we noted that FMOD treatment significantly increased the mean *F*_*D*_ values of the wounds, and even approximated *F*_*D*_ values of unwounded skin when quantified at 8 weeks post-injury (Figure 2c and e). FMOD treatment also decreased the mean *L* values to a similar level of the *L* values of unwounded skin at 8 weeks post-injury, which indicates a notably more organized distribution of uniformly sized collagen fibers in the FMOD-treated wounds (Figure 2c and f). Importantly, the reduced scar size in FMOD-treated wounds did not occur at the expense of diminished tensile strength. In fact, tensile strength was significantly increased in all FMOD-treated groups, relative to controls, at 4 and 8 weeks post-injury (Figure 2g). Overall, these data demonstrate that FMOD application reduced scar size, increased tensile strength, and improved dermal collagen architecture organization in preclinical animal models.

### FMOD markedly alters gene expression during wound healing

**Figure 3:**
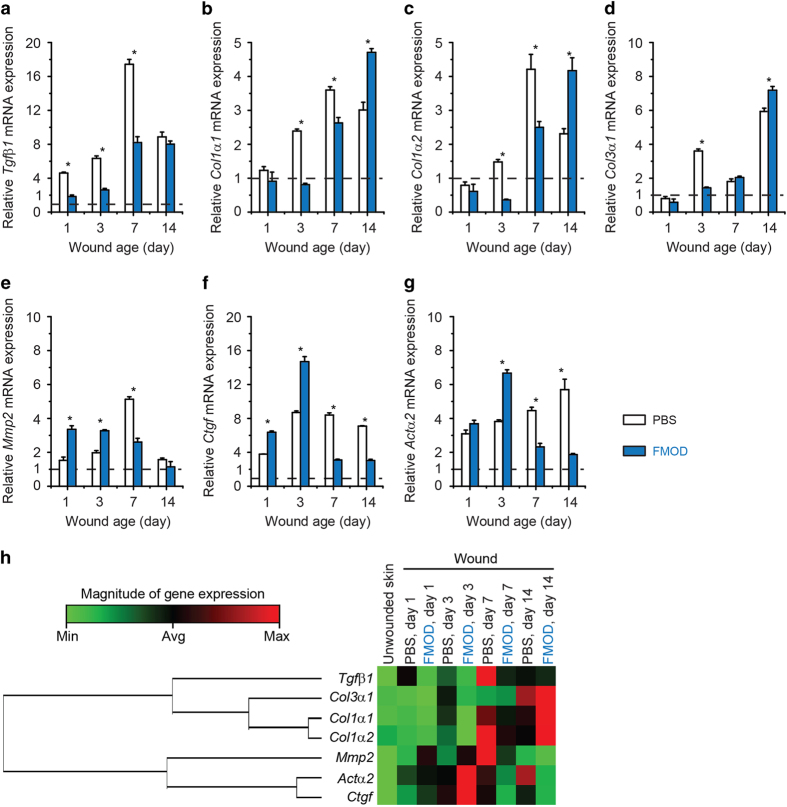
FMOD administration altered the expression of TGFβ1-related genes during adult rat cutaneous wound healing. Expression of *Tgfβ1* (**a**), *Col1α1* (**b**), *Col1α2* (**c**), *Col3α1* (**d**), *Mmp2* (**e**), *Ctgf* (**f**), and *Actα2* (**g**) was compared between PBS (control) and 2.0 mg ml^−1^ FMOD-treated adult rat cutaneous wounds. Clustergram plot further indicated that FMOD diversely modulated the expression of TGFβ1-related genes during adult rat cutaneous wound healing (**h**). A two-sample *t-*test was used for statistical analysis. Data were normalized to unwounded skin (dashed lines), and are shown as mean±the standard deviation. *N*=3 wounds from 3 animals pooled for each sample (9 wounds from 9 animals in total). *P<0.05.

To further understand the molecular basis underlying FMOD’s wound healing enhancing effects, a RT^2^ profiler PCR array was utilized to screen for FMOD-responsive genes during the adult rat wound healing period (Supplementary Figure 5). We previously observed an inverse correlation between Fmod and Tgfβ1 levels during fetal wound healing in rodents. High Fmod levels correlated with decreased Tgfβ1 expression and scarless repair, while low Fmod levels correlated with increased Tgfβ1 expression and scar formation.^17^ Corresponding to our fetal rodent data, FMOD application to adult rats reduced early (days 1, 3, and 7) *Tgfβ1* transcripts (Figure 3a). Not surprisingly, FMOD treatment also decreased expression of TGFβ1-responsive genes that encode fibrotic collagen, specifically collagen (*Col)1α1* and *Col1α2* (days 3 and 7; Figure 3b and c), and *Col3α1* (day 3; Figure 3d). These collagen genes contribute to excessive ECM production and scar formation at early time points post-injury, and are subsequently upregulated during the remodeling phase of wound healing (day 14). Somewhat unexpectedly, FMOD application elevated another subset of TGFβ1-responsive genes implicated in ECM remodeling, fibroblast migration/invasion, and myofibroblast differentiation/contraction;^4,9,36^ all of which are events that occur before wound closure, typically by post-injury day 5. These genes include matrix metalloproteinase 2 (*Mmp2*; Figure 3e), *Ctgf* (Figure 3f), and *Actα2* (encoding α-Sma; Figure 3g). After complete wound re-epithelialization, these genes were noticeably downregulated in the FMOD-treated groups. Consequently, FMOD application appears to be associated with both the early upregulation of TGFβ1-responsive genes that promote cellular migration and myofibroblastic conversion, and the early downregulation of TGFβ1-responsive genes that promote fibrotic ECM deposition and scarring (Figure 3h).

### FMOD modulates the behavior of adult dermal fibroblasts

**Figure 4:**
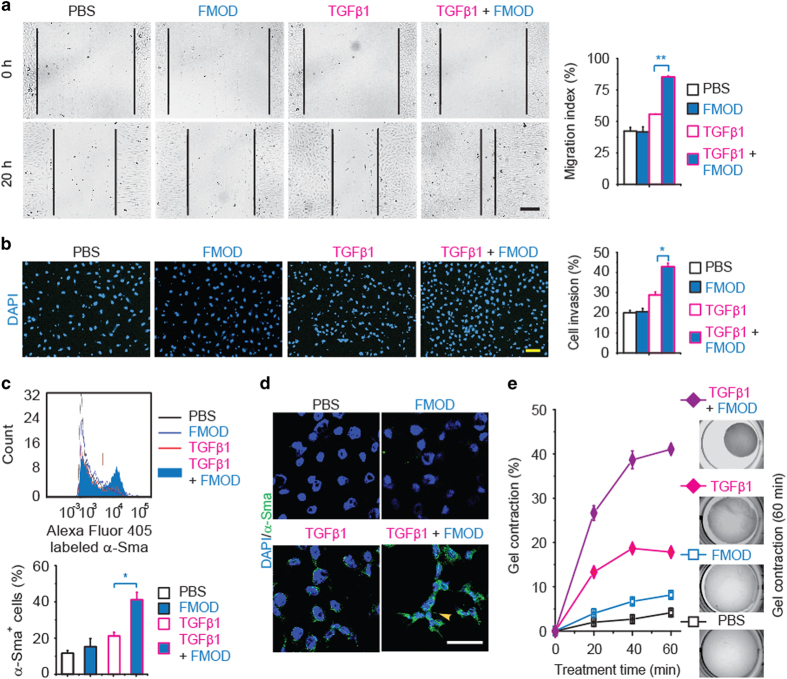
FMOD enhanced TGFβ1-induced rat dermal fibroblast (RDF) migration and invasion, and myofibroblast conversion and contraction. FMOD+TGFβ1 significantly promoted TGFβ1-mediated RDF migration (**a**) and invasion through collagen matrices (**b**). Flow cytometry tests revealed that FMOD significantly enhanced TGFβ1-induced rat dermal fibroblast-myofibroblast conversion (**c**), which corresponds with immunofluorescent staining (**d**). Interestingly, after 48 h treatment, α-Sma^+^ myofibroblasts formed stress fibers (yellow arrows) in the FMOD+TGFβ1 group. Additionally, FMOD+TGFβ1 significantly stimulated TGFβ1-mediated myofibroblast contraction in collagen gel (**e**). Dosages: TGFβ1 (100 pm) and FMOD (200 nM). Scale bar=100 μm (**a**, **b**), or 50 μm (**d**). Two-sample *t-*test was used for statistical analysis, and data are shown as mean±the standard deviation *N*=4 (**a**, **b**), 3 (**c**), or 6 (**e**). *P<0.05, **P<0.01.

As fibroblasts are the predominant cell type required for dermal repair, we tested FMOD’s effects on adult RDFs. First, up to 200 nm FMOD alone did not affect RDF proliferation; however, FMOD significantly stimulated RDF proliferation in the presence of TGFβ1 (Supplementary Figure 6). Second, while FMOD alone did not increase cell motility, FMOD+TGFβ1 significantly promoted RDF migration and invasion (Figure 4a and b) and markedly enhanced myofibroblast differentiation and contractility, as shown by α-Sma staining (Figure 4c and d). Quantitative analysis of 3D collagen gel contraction studies confirmed an increased contractility of FMOD+TGFβ1-induced myofibroblasts, relative to contractility induced by TGFβ1 alone (Figure 4e). In contrast, FMOD alone temporarily decreased RDF *Col3α1* expression in the absence of exogenous TGFβ1 administration (Supplementary Figure 7a) and significantly inhibited the ability of TGFβ1 to stimulate RDF *Col1α1* and *Col1α2* transcription (Supplementary Figures 7b and c). Additionally, FMOD temporarily enhanced RDF Mmp2 expression, regardless the existence of TGFβ1 (Supplementary Figures 7d and e). Overall, FMOD-treated adult RDFs exhibited increased migration, and myofibroblast differentiation and contractility, with less fibrotic ECM production. These findings paralleled the reduced scar formation and accelerated wound closure seen in FMOD-treated adult rat wounds *in vivo* (Figure 1).

**Figure 5:**
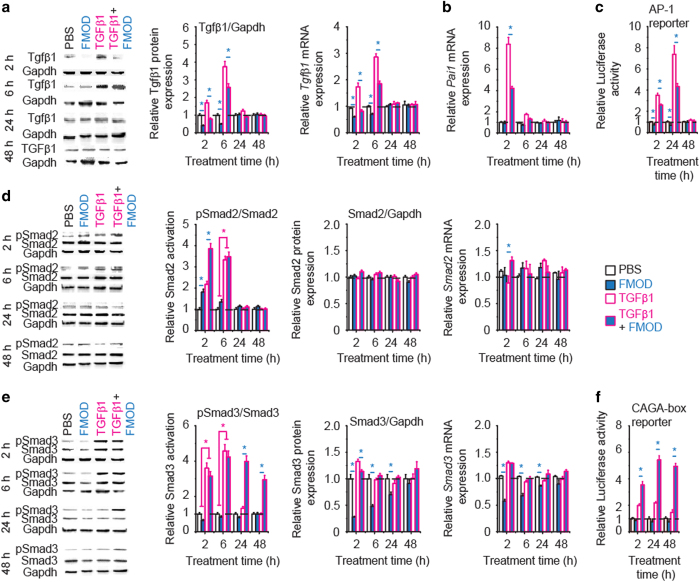
FMOD delicately orchestrated Tgfβ1 signal transduction in adult RDFs. Western blotting and qRT-PCR revealed that FMOD reduced Tgfβ1 expression and Tgfβ1 auto-induction (**a**), and inhibited TGFβ1-dependent *Pai1* transcription in adult RDFs (**b**). Using an AP-1-luciferase reporter system, we further revealed that FMOD significantly downregulated AP-1-mediated non-canonical TGFβ1 signal transduction (which predominately regulates TGFβ1 auto-induction and *Pai1* expression) up to 24 h post-treatment (**c**). Meanwhile, FMOD stimulated adult RDF Smad2 phosphorylation/activation when measured over a short amount of time (2 h) in the presence or absence of TGFβ1, but did not have an obvious effect on Smad2 expression (**d**). Although FMOD alone downregulated initial Smad3 expression up to 24 h post-treatment, when combined with TGFβ1, FMOD did not affect Smad3 expression in the 48-h experiment period, but it significantly prolonged TGFβ1-responsive Smad3 phosphorylation/activation (**e**), which was confirmed using a CAGA-box (specific for SMAD3 signal transduction) Luciferase reporter system (**f**). Dosages: TGFβ1 (100 pM) and FMOD (200 nM). A two-sample *t-*test was used for statistical analysis. Data were normalized to untreated RDFs at time 0 (dashed lines) and shown as mean±the standard deviation. *N*=3 (**a**, **b**, **d**, **e**) or 5 (**c**, **f**). *P<0.05; a blue star indicates the significance in comparison with FMOD administration, and a magenta star indicates the significance in comparison with TGFβ1 administration.

Consistent with our *in vivo* findings (Figure 3a) and previous studies,^17,19,20^ FMOD strongly suppressed Tgfβ1 expression and auto-induction in adult RDFs (Figure 5a). FMOD also significantly repressed TGFβ1-induced expression of plasminogen activator inhibitor (Pai)1, a well-known pro-fibrotic molecule that contributes to excessive accumulation of collagen,^37^ in adult RDFs (Figure 5b). The downregulation of Tgfβ1 and Pai1 in FMOD-treated RDFs could be attributed to FMOD significantly decreasing the AP-1-mediated TGFβ1 signal transduction (Figure 5c), which has been reported to control TGFβ1 auto-induction and PAI1 expression.^37,38^ In contrast, FMOD treatment promoted rapid Smad2 phosphorylation/activation in adult RDFs (Figure 5d), which correlates with Mmp2 expression. Since SMAD2, but not SMAD3, has been reported to regulate MMP2 expression,^39^ these findings suggest that FMOD-induced phosphorylation of SMAD2 may be responsible for the transient upregulation of Mmp2. Notably, FMOD stimulation of Smad2 signaling occurred concomitantly with FMOD repression of AP-1 activation. Understanding that AP-1 promotes TGFβ1 auto-induction,^38^ while SMAD2 regulates MMP2 expression,^39^ may explain the seemingly paradoxical coupling of decreased TGFβ1 expression while upregulating TGFβ1 downstream target MMP2 in earlier human dermal fibroblasts studies after adenovirus-mediated FMOD overexpression.^40^

**Figure 6:**
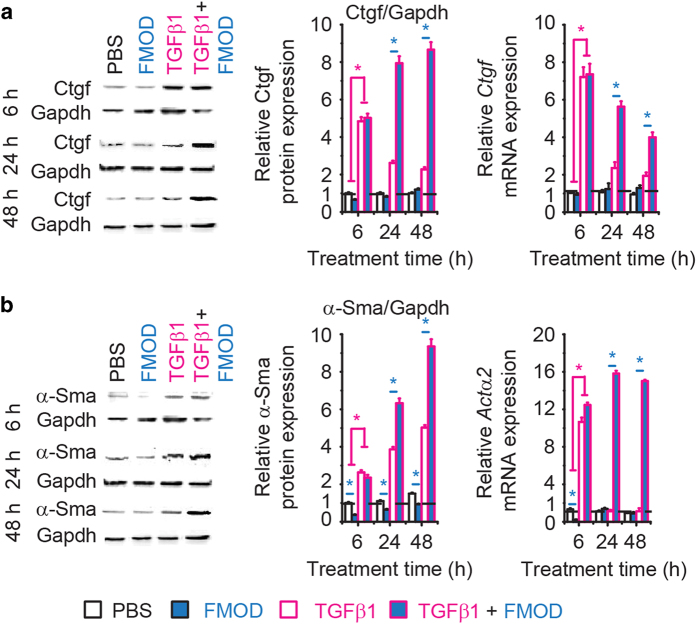
FMOD enhanced TGFβ1-responsive Ctgf and α-Sma expression in adult RDFs. In the presence of TGFβ1, FMOD significantly increased expression of Ctgf (**a**) and a-Sma (**b**) of RDFs. Dosages: TGFβ1 (100 pM) and FMOD (200 nm). A two-sample *t-*test was used for statistical analysis. Data were normalized to untreated RDFs at time 0 (dashed lines) and shown as mean±the standard deviation. *N*=3. *P<0.05; a blue star indicates the significance in comparison with FMOD administration, and a magenta star indicates the significance in comparison with TGFβ1 administration.

With respect to Smad3, FMOD alone slightly inhibited its expression in RDFs, while FMOD+TGFβ1 combination significantly prolonged Smad3 phosphorylation/activation without altering its gene or protein expression (Figure 5e). Using a luciferase-reporter system specific for SMAD3 signal transduction (that is, CAGA-box reporter), we confirmed that FMOD prolonged TGFβ1-induced Smad3 signaling in RDFs *in vitro* (Figure 5f). Similarly, increased phosphorylated SMAD3 was detected in FMOD-treated adult rat wounds at day 3 post-injury (Supplementary Figure 8). FMOD-mediated increase in Smad3 signal transduction in FMOD+TGFβ1-treated RDFs could explain the elevated expression of Smad3-dependent molecules Ctgf and α-Sma (Figure 6), which are known to significantly augment migration and contraction.^9,36^ Furthermore, two specific inhibitors of SMAD3-signal transduction, naringenin and SIS3, were used to attenuate Smad3-signaling in adult RDFs. Both inhibitors diminished the positive effects of FMOD on Ctgf and α-Sma expression, migration, invasion, and contraction of RDFs (Supplementary Figures 9 and 10). Importantly, co-administration of specific inhibitors of SMAD3-signal transduction with FMOD completely eliminates the ability of FMOD to reduce scar size and increase tensile strength in adult rat wounds (Figure 1). Comprehensively, these results confirmed that FMOD’s pro-migratory and pro-contractility effects that reduce scar size and increase scar strength require intact TGFβ1-responsive SMAD3 signaling activation.

## Discussion

In this study, we demonstrate that intradermal recombinant human FMOD injection significantly reduced scar size by 50–70% in adult animal wounds of loose-skin rodent and fixed-skin porcine models.^41^ Importantly, FMOD-mediated improvements in wound healing are accompanied by a marked increase in wound tensile strength, which represents a potential significant improvement over the current standards of care. For example, corticosteroids, used in the current standard of care, have been found to impair wound healing and decrease scar strength.^42,43^ We believe our finding provides the first successful case demonstrating feasibility in shifting the paradigm of reducing scar formation from multifarious therapies involving cytokine-growth factor-steroid cocktails and laser treatments to a simple, biological approach using native ECM molecule(s).

**Figure 7:**
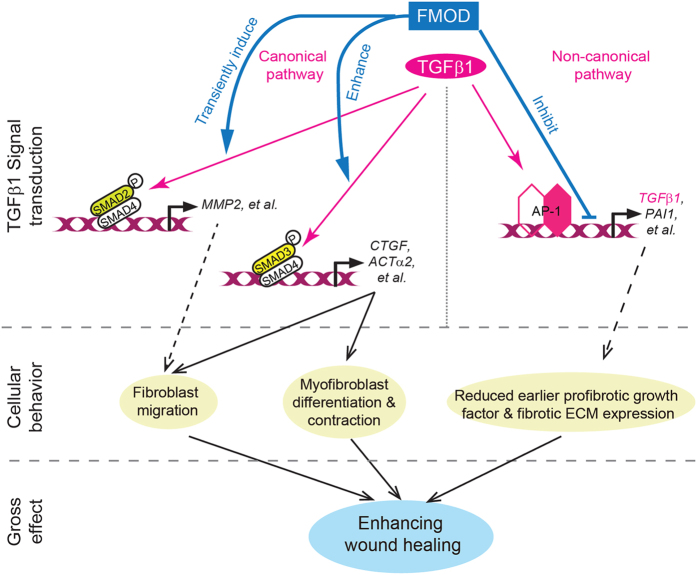
FMOD delicately orchestrated TGFβ1-signaling during adult cutaneous wound healing. Rather than indiscriminately blocking TGFβ1 signals, FMOD selectively regulated TGFβ1 bioactivities in a signal transduction pathway-dependent manner.

Our current study elucidates the critical function of FMOD in regulating wound healing. Much to our surprise, FMOD selectively augments early pro-migratory and pro-contractile canonical TGFβ1 signaling to decrease scar size and increase wound tissue tensile strength, while simultaneously repressing TGFβ-stimulated fibrotic gene expression to decrease scar tissue formation (Figure 7 and Supplementary Video 1). For the first time, we can predictably uncouple the beneficial pro-migration/contraction effects of TGFβ1 that promote faster and stronger wound closure from the pro-fibrotic effects of TGFβ1 that promote excessive scarring. Future studies will further examine how FMOD delicately orchestrates TGFβ signal transduction, such as uncoupling the canonical and non-canonical TGFβ1 signaling pathways.

Importantly, we also demonstrate the critical role that the FMOD-TGFβ1-CTGF nexus plays in rapid cell migration and myofibroblast function. TGFβ1-CTGF signal transduction, which has been thought of as a culprit contributing to excessive scar formation, can now be considered as part of the solution when in the presence of FMOD. This paradigm-shifting concept fundamentally challenges the current drug development focus on ‘low TGFβ1/CTGF’ and/or ‘high TGFβ3’ targets for minimizing human cutaneous scarring. Indeed, our data show that increased TGFβ1-mediated CTGF signaling in the early period after skin injury is actually beneficial for: (1) rapid fibroblast migration (2), rapid fibroblast to myofibroblast conversion (3), increased myofibroblast contractility to promote wound closure, and (4) increased wound tensile strength. Publically available data appear to validate our hypothesis that indiscriminate blockade of TGFβ1 and its downstream mediator CTGF, especially at early stage of wound healing, is not optimal for human scar reduction. TGFβ1-neutralizing antibodies and peptides have failed to reduce scar formation in preclinical animal studies.^7,8^ Moreover, clinical trials appear to be halted for PF-0673871/EXC001, an antisense oligonucleotide product targeting CTGF,^10^ while recent Phase 2 clinical trial results from RXI Pharmaceuticals show that application of RXI-109—another RNA interference (RNAi) product targeting CTGF—starting 2 weeks post-surgery leads to better scar reduction scores than an application immediately after injury.^11^ Interestingly, our present work that underscores the importance of early fibroblast migration, and myofibroblast conversion and contraction, for optimal repair—and FMOD’s role in these processes—may also explain the lack of efficacy observed in Phase 3 clinical trials for Juvista (Avotermin, recombinant human TGFβ3 by Renovo, Manchester, UK).^44^ Although TGFβ3 has been widely described as a potent anti-fibrotic molecule^4^ initially identified through scarless fetal wound healing studies, a lesser known effect of TGFβ3 is its well-documented inhibition of adult dermal fibroblast proliferation and migration.^45,46^ In agreement with this effect, we found TGFβ3 levels to be dramatically elevated in adult *Fmod*^*−/−*^ mouse wounds that exhibited severely delayed fibroblast migration and granulation tissue formation, which ultimately healed with increased scar size.^19^ Moreover, we showed that TGFβ3 addition markedly inhibited fibroblast migration, and that FMOD could directly reverse this effect *in vitro* and *in vivo* to restore fibroblast migration and reduce scar size.^19^

Recent discoveries highlight how SLRP functions extend well beyond their originally described roles in ECM structural support.^18,47^ SLRPs are now widely recognized as key regulators of intracellular signal cascades that govern multiple processes, such as angiogenesis and cell fate determination.^21,25,30,48,49^ Our previous studies identified that FMOD alone can reprogram somatic cells to a multipotent state under a serum-free situation *in vitro*.^25,30^ We also found that FMOD could restore the fetal-type scarless healing with hair follicle regeneration in late-gestation fetal wounds, which normally heal but form a scar.^16,17^ In the current study, we observed that FMOD treatment results in adult fibroblasts and wounds bearing several striking similarities to the attributes of fetal fibroblasts and fetal wounds: first, like fetal wounds,^15^ FMOD treatment to adults wounds causes reduced and more transient TGFβ1 expression; second, like fetal wounds,^50^ FMOD treatment induces high levels of SMAD2 and SMAD3 phosphorylation, and low levels of several fibrosis-associated targets; third, like fetal fibroblasts,^51^ FMOD treatment results in a more migratory and contractile phenotype; fourth, like fetal fibroblasts, FMOD treatment exhibits higher TGFβ1-stimulated CTGF expression levels for increased myofibroblast differentiation and contraction;^52^ and lastly, much like fetal wounds,^53^ the markedly reduced *Actα2* expression at days 7 and 14 post-injury (Figure 3g) in the FMOD-treated groups suggests that FMOD treatment results in more rapid myofibroblast clearance from the wound. Taken together, FMOD administration in adult wound models elicits a similar phenotype to fetal wounds at the molecular, cellular, and gross morphological levels. This unique response of living systems to FMOD, an extracellular SLRP molecule involved in collagen fibrillogenesis,^18^ has not been previously described. Considering that the expression of FMOD significantly decreased in senescent fibroblasts,^54,55^ the fetal-like phenotypes induced by FMOD in adult dermal fibroblasts *in vitro* and adult cutaneous wounds *in vivo,* as demonstrated in the current study, aptly qualifies FMOD as a matricellular regulator with diverse functions beyond its structural support roles, and may open a new avenue to develop regenerative medicines that ‘turn back the clock’ for more youthful living.

However, administration of FMOD was not able to fully stimulate a fetal-type scarless healing with hair follicle regeneration or significantly increase the type III collagen content in adult wound models, which incorporate the complexity of the wound healing process and illustrates that other molecules, in addition to FMOD and TGFβ, are also important for scarring. For instance, recent human studies suggest that single-nucleotide polymorphisms in several loci or genes are also associated with hypertrophic scar and keloid formation in different populations.^56–59^ Therefore, further investigation is required to entirely understand the nature of wound healing.

In summary, we systematically demonstrated that FMOD administration potently reduced scar size, improved scar appearance, and increased scar strength in both an adult rodent model and a porcine model to simulate human wound healing. With pig skin being recognized by the US FDA as the closest animal equivalent to human skin,^23,24^ there is high translational potential and applicability of our current findings to human patients. To examine whether FMOD’s significant effects on cell migration and contraction can also translate to new therapies for clinical conditions associated with inadequate fibroplasia, such as poor healing from illness, medications or chronic wounds, the preclinical efficacy of FMOD should be validated in splinting excisional animal models.^22,60^ In addition, in order to apply FMOD clinically, Good Manufacturing Practice (GMP)-compliant high-purity FMOD production should be established, and the safety of FMOD should be fully assessed in advance of clinical trials.
