# Supplementary material for: Fibromodulin reduces scar formation in adult cutaneous wounds by eliciting a fetal-like phenotype
Source: Signal Transduct Target Ther. 2017 Oct 13;2:17050–. doi: 10.1038/sigtrans.2017.50 (PMC5661627; doi:10.1038/sigtrans.2017.50)
Supplement: Supplementary Information [file sigtrans201750-s1.docx]

**Supplementary Information**

**Supplementary Table 1. Antibodies.**

| Antigen | Species | Company |
| --- | --- | --- |
| TGFβ1 | R, P^1^, *Xenopus*, M^1^, H^1^ | Santa Cruz Biotech. |
| GAPDH | R, M, H | Santa Cruz Biotech. |
| pSMAD2^2^ | R, M, H | Abcam Inc. |
| SMAD2 | R, M, H | Abcam Inc. |
| pSMAD3^3^ | Ch^1^, C^1^, P, R, M, H | Abcam Inc. |
| SMAD3 | R, M, H | Abcam Inc. |
| CTGF | R, M, H | Santa Cruz Biotech. |
| α-SMA | R, M, H, Ch, C, P | Abcam Inc. |

^1^ R, rat; P, pig; M, mouse; H, human; Ch, chicken; and C, cow.

^2^ pSMAD2: phosphorylated SMAD2 (phospho S467)

^3^ pSMAD3: phosphorylated SMAD3 (phospho S423 + S425)

**Supplementary Table 2. Primers and probes for qRT-PCR.**

*Taqman^®^ Gene Expression Assays purchased from Applied Biosystems^TM^*

| Species | Gene name | Ref. Seq. |
| --- | --- | --- |
| Rat | *Gapdh* | NM_017008.3 |
| Rat | *Tgfβ1* | NM_021578.2 |
| Rat | *Smad2* | NM_019191.1 |
| Rat | *Smad3* | NM_013095.2 |
| Rat | *Ctgf* | NM_022266.2 |
| Rat | *Actα2* | NM_031004.2 |
| Rat | *Mmp2* | NM_031054.2 |
| Rat | *Col1α1* | NM_053304.1 |
| Rat | *Col1α2* | NM_053356.1 |
| Rat | *Col3α1* | NM_032085.1 |
| Mouse | *Gapdh* | NM_008084.2 |
| Mouse | *Tgfβ1* | NM_011577.1 |

**Supplementary Table 2 (continued)**:

*Primers for SYBR^®^ Green-based qRT-PCR of rat genes (Invitrogen)*

| Gene | Primer sequence | |
| --- | --- | --- |
| *Gapdh* | Sense | 5’-AGGACCAGGTTGTCTCCTGT-3’ |
|  | Antisense | 5’-TTACTCCTTGGAGGCCATGT-3’ |
| *Pai1* | Sense | 5’-CTTTATCCTGGGTCTCCCTG-3’ |
|  | Antisense | 5’-TGATGCCTCCCTGACATACA-3’ |

**Supplementary figure legends**

**Supplementary Fig. 1. Criteria of gross appearance assessment of adult Yorkshire pig wounds by VAS were established.**

On a standard sample obtained 2 weeks post-injury from adult female Yorkshire pigs, a value between 0 (unwounded pig skin) and 100 (poorly healed wounds) was given, with lower values indicating subjective improvements in scar appearance and reduction in scar size (**a**). Three MDs scored each scar in a randomized, double-blind fashion, and the scores were averaged to generate a final VAS score as shown in this example sample obtained at 8 weeks post-injury (**b**).

**Supplementary Fig. 2. Multiple staining methods were used to analyze the scar size of adult Yorkshire pig wounds.**

Compared with hematoxylin and eosin (H&E) and Masson’s trichrome staining, picrosirius red staining (PSR)-coupled polarized light microscopy (PLM) distinguished clearer boundaries between scar tissue (S; right) and unwounded normal dermis (D, left) as evidenced at higher magnifications. The resenting wound was treated with PBS and harvested at 4 weeks post-injury. Scars are outlined by cyan lines, while dermis layers are outlined by dashed magenta lines on PSR-PLM photographs. Scale bar = 500 μm (red), or 100 μm (white).

**Supplementary Fig. 3.** **RDFs were tested negatively for mycoplasma contamination.**

Mycoplasma contamination was tested by Universal Mycoplasma Detection Kit (ATCC). M: 100 bp DNA ladder with highlighted band at 500 bp; lane 1, positive control; lane 2, positive control + RDF sample; lane 3, RDF sample; and lane 4, negative control.

**Supplementary Fig. 4.** **FMOD administration reduced scar size in adult WT and *Fmod^-/-^* mouse cutaneous wounds.**

H&E staining of PBS or 0.4 mg/ml FMOD-treated adult WT and *Fmod^-/-^* mouse wounds at day 14 post-injury (**a**). Scar size differences quantified by Scar Index (**b**). Scars are outlined by yellow lines. Scale bar = 200 μm. Mann-Whitney test was used for statistical analysis. Mean values were presented. N = 14-32 wounds from 14-16 animals, respectively. *, *P* < 0.05; a red star indicates significance in comparison with *Fmod^-/-^* mouse wounds, and a blue star indicates significance in comparison with exogenous FMOD administration.

**Supplementary Fig. 5. FMOD administration markedly altered the expression of wound repair-related genes during adult rat wound healing.**

The clustergram displays the hierarchical clustering of the expressions of 84 key genes central to the rat wound healing response. N = 3 wounds from 3 animals pooled for each sample (9 wounds from 9 animals in total).

**Supplementary Fig. 6.** **FMOD promoted adult rat dermal fibroblast (RDF) proliferation in the presence of TGFβ1.**

FMOD alone did not significantly affect adult RDF proliferation when applied at concentrations below 200 nM. At higher concentrations (400 nM and 800 nM), FMOD application resulted in decreased RDF proliferation. Although TGFβ1 alone inhibited RDF proliferation, FMOD significantly enhanced RDF proliferation in the presence of TGFβ1. Dosages: TGFβ1 (100 pM) and FMOD (0-800 nM). Data were normalized to untreated RDFs (dashed lines). Mean and standard deviation values were obtained using a two-sample *t*-test. N = 6. *, *P* < 0.05; a black star indicates significance in comparison with untreated RDFs, and a magenta star indicates significance in comparison with TGFβ1 alone.

**Supplementary Fig. 7.** **FMOD decreased fibrotic ECM expression while temporarily increasing Mmp2 levels in adult RDFs.**

FMOD temporarily reduced type III collagen gene (*Col3α1*) expression in RDFs in the absence of TGFβ1 (**a**). Although FMOD alone did not discernably affect expression of RDF type I collagen genes *Col1α1* (**b**) and *Col1α2* (**c**) in the absence of TGFβ1, FMOD significantly downregulated TGFβ1-induced transcription of *Col1α1* and *Col1α2*. On the other hand, qRT-PCR (**d**) and ELISA (**e**) revealed that FMOD temporarily enhanced Mmp2 expression in RDFs. Dosages: TGFβ1 (100 pM) and FMOD (200 nM). Data were normalized to untreated RDFs at time 0 (dashed lines; **a-d**) and shown as mean ± the standard deviation, obtained through a two-sample *t-*test. N = 3. *, *P* < 0.05; a blue star indicates the significance in comparison with FMOD administration, and a magenta star indicates the significance in comparison with TGFβ1 administration.

**Supplementary Fig. 8. FMOD-treated adult rat wounds had increased activated/phosphorylated Smad3 (pSmad3)-positive fibroblasts at the wound edge, 3 days post-injury when compared with PBS-treated controls.**

Scale bar = 200 μm (blue) or 100 μm (black).

**Supplementary Fig. 9. Naringenin significantly reduced Smad3-signal transduction and eliminated the pro-migration/contraction effects of FMOD in adult RDFs.**

Western blotting and qRT-PCR analyses confirmed that naringenin (which selectively inhibits the expression of SMAD3, but not SMAD2, SMAD4 or SMAD7) inhibited Smad3 expression in RDFs in the presence or absence of TGFβ1 and/or FMOD (**a**). Meanwhile, naringenin markedly blocked the enhancement of FMOD on TGFβ1-responsible expression of Ctgf (**b**) and α-Sma (**c**), and completely eliminated the pro-migration/contraction of FMOD on RDF migration (**d**), invasion through collagen matrices (**e**), and contraction in collagen gel (**f**). Dosages: TGFβ1 (100 pM), FMOD (200 nM), and naringenin (50 μM). Scale bar = 100 μm. Data were normalized to untreated RDFs at time 0 (dashed lines; **a-c**) and shown as mean ± the standard deviation, obtained through a two-sample *t-*test. N = 3 (**a-c**), or 4 (**d**, **e**), or 6 (**f**). *, *P* < 0.05; a blue star indicates the significance in comparison with FMOD administration, and a magenta star indicates the significance in comparison with TGFβ1 administration.

**Supplementary Fig. 10. SIS3 significantly reduced Smad3-signal transduction and eliminated the pro-migration/contraction effects of FMOD in adult RDFs.**

SIS3 (which attenuates phosphorylation of SMAD3 but not SMAD2) blocked Smad3 phosphorylation/activation in adult RDFs, without evident effects on Smad3 expression (**a**). SIS3 markedly blocked enhancement of FMOD on TGFβ1-responsible expression of Ctgf (**b**) and α-Sma (**c**), and completely eliminated the pro-migration/contraction of FMOD on RDF migration (**d**), invasion through collagen matrices (**e**), and contraction in collagen gel (**f**). Dosages: TGFβ1 (100 pM), FMOD (200 nM), and SIS3 (5 μM). Scale bar = 100 μm. Data were normalized to untreated RDFs at time 0 (dashed lines; **a-c**) and shown as mean ± the standard diviation, obtained through a two-sample *t-*test.. N = 3 (**a-c**), or 4 (**d**, **e**), or 6 (**f**). *, *P* < 0.05; a blue star indicates the significance in comparison with FMOD administration, and a magenta star indicates the significance in comparison with TGFβ1 administration.

**Supplementary Video 1: FMOD plays unique roles in regulating cutaneous wound healing.**

In this study, we demonstrate that application of FMOD leads to enhanced fibroblast migration and myofibroblast maturation and contraction, which results in reduced scar size, increased tensile wound strength, and improved gross appearance.
