# Supplementary figures and images for: Fibromodulin reduces scar formation in adult cutaneous wounds by eliciting a fetal-like phenotype
Source: Signal Transduct Target Ther. 2017 Oct 13;2:17050–. doi: 10.1038/sigtrans.2017.50 (PMC5661627; doi:10.1038/sigtrans.2017.50)

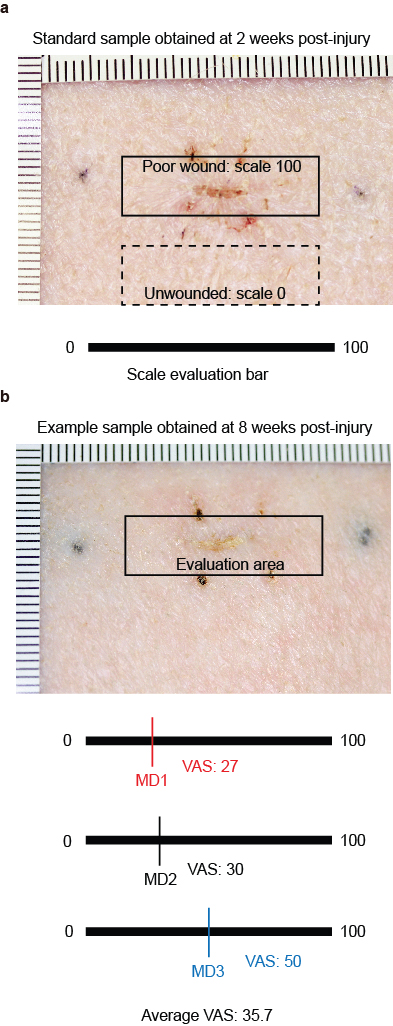

Supplement: Supplementary Figure 1 [file sigtrans201750-s2.jpg]

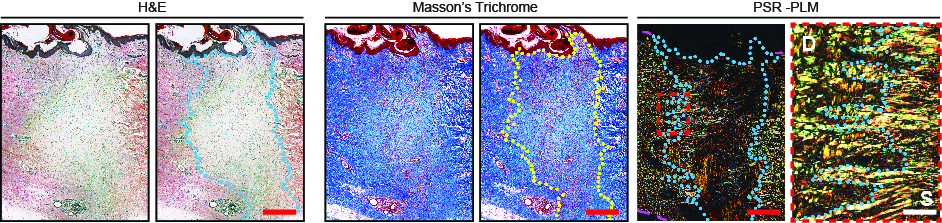

Supplement: Supplementary Figure 2 [file sigtrans201750-s3.jpg]

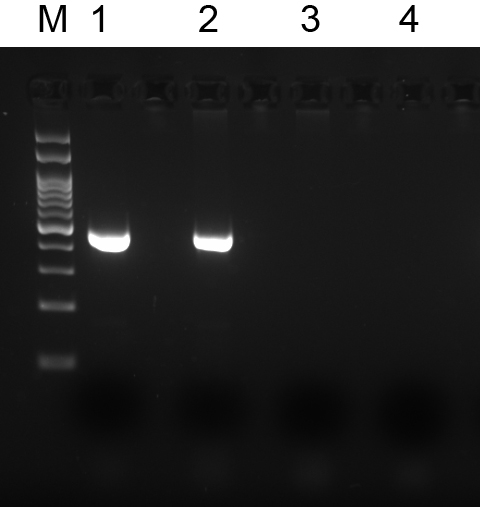

Supplement: Supplementary Figure 3 [file sigtrans201750-s4.jpg]

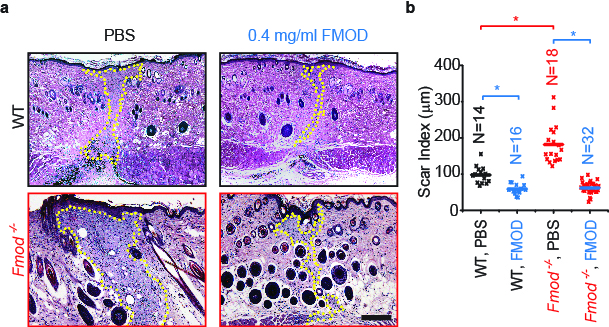

Supplement: Supplementary Figure 4 [file sigtrans201750-s5.jpg]

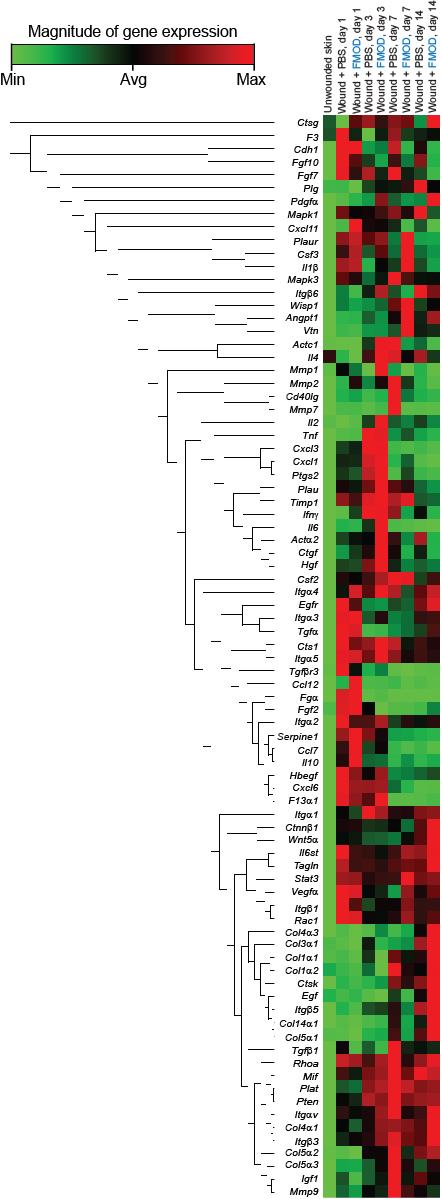

Supplement: Supplementary Figure 5 [file sigtrans201750-s6.jpg]

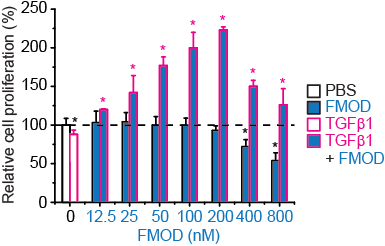

Supplement: Supplementary Figure 6 [file sigtrans201750-s7.jpg]

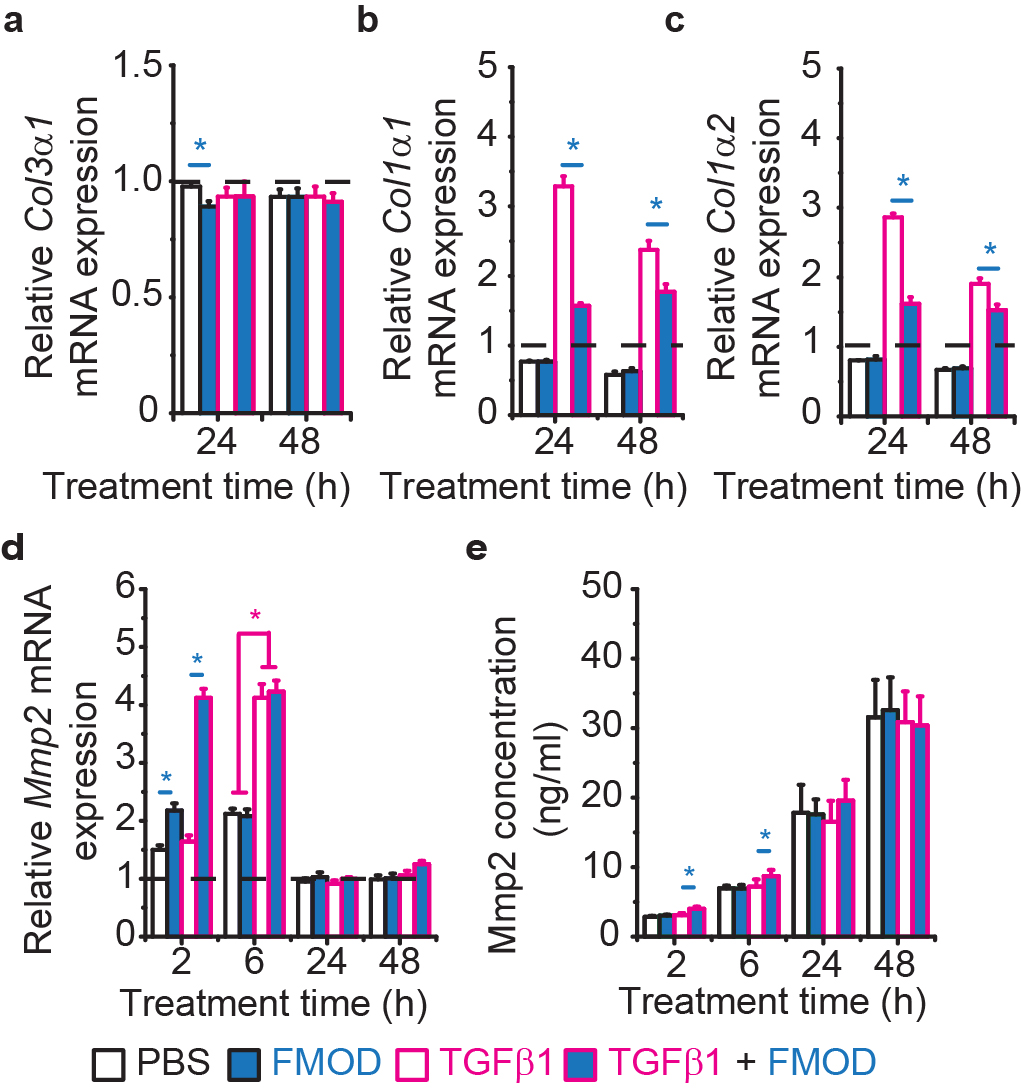

Supplement: Supplementary Figure 7 [file sigtrans201750-s8.jpg]

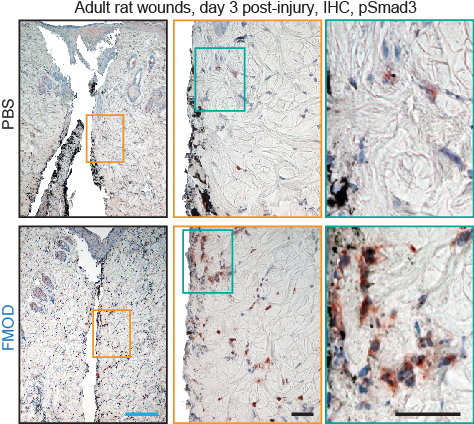

Supplement: Supplementary Figure 8 [file sigtrans201750-s9.jpg]

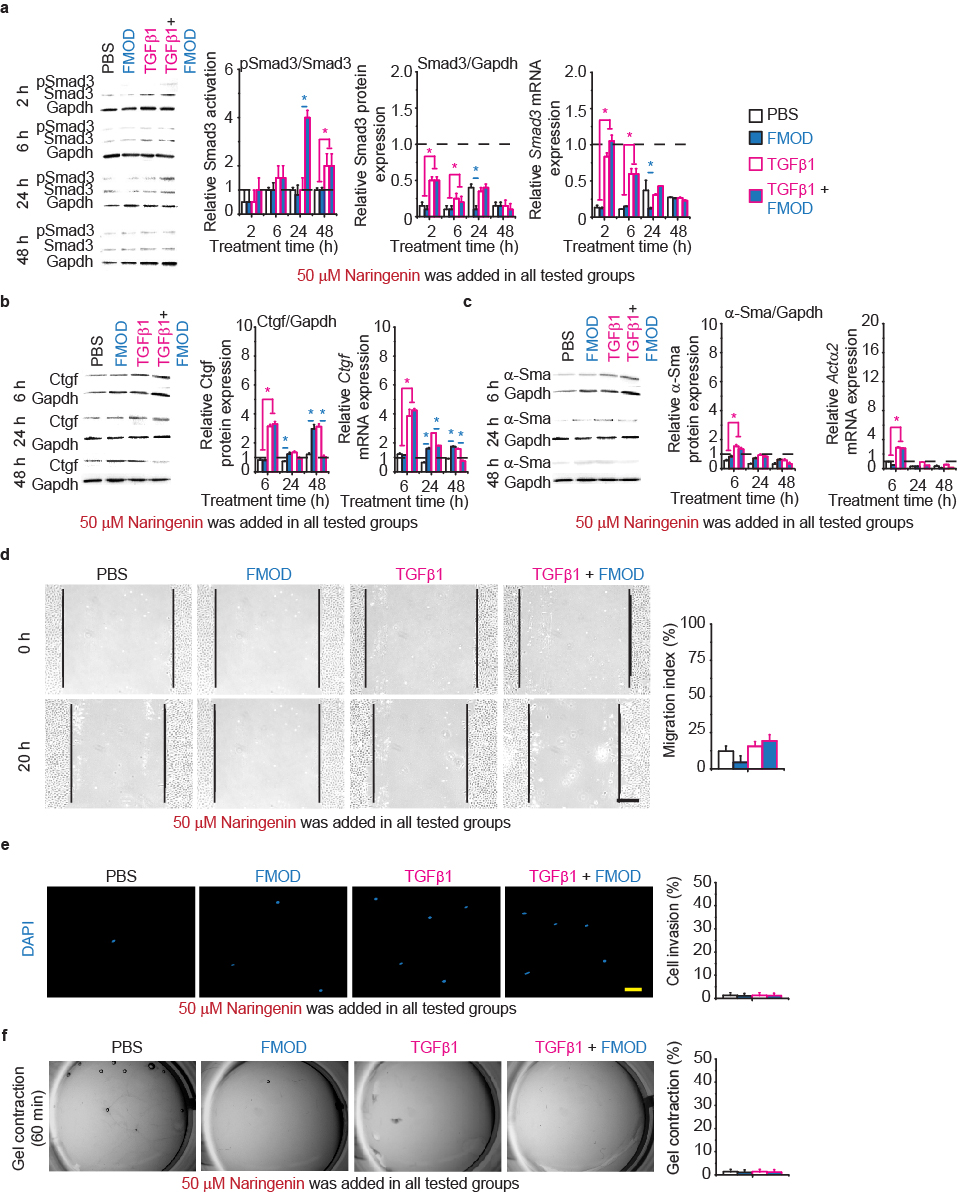

Supplement: Supplementary Figure 9 [file sigtrans201750-s10.jpg]

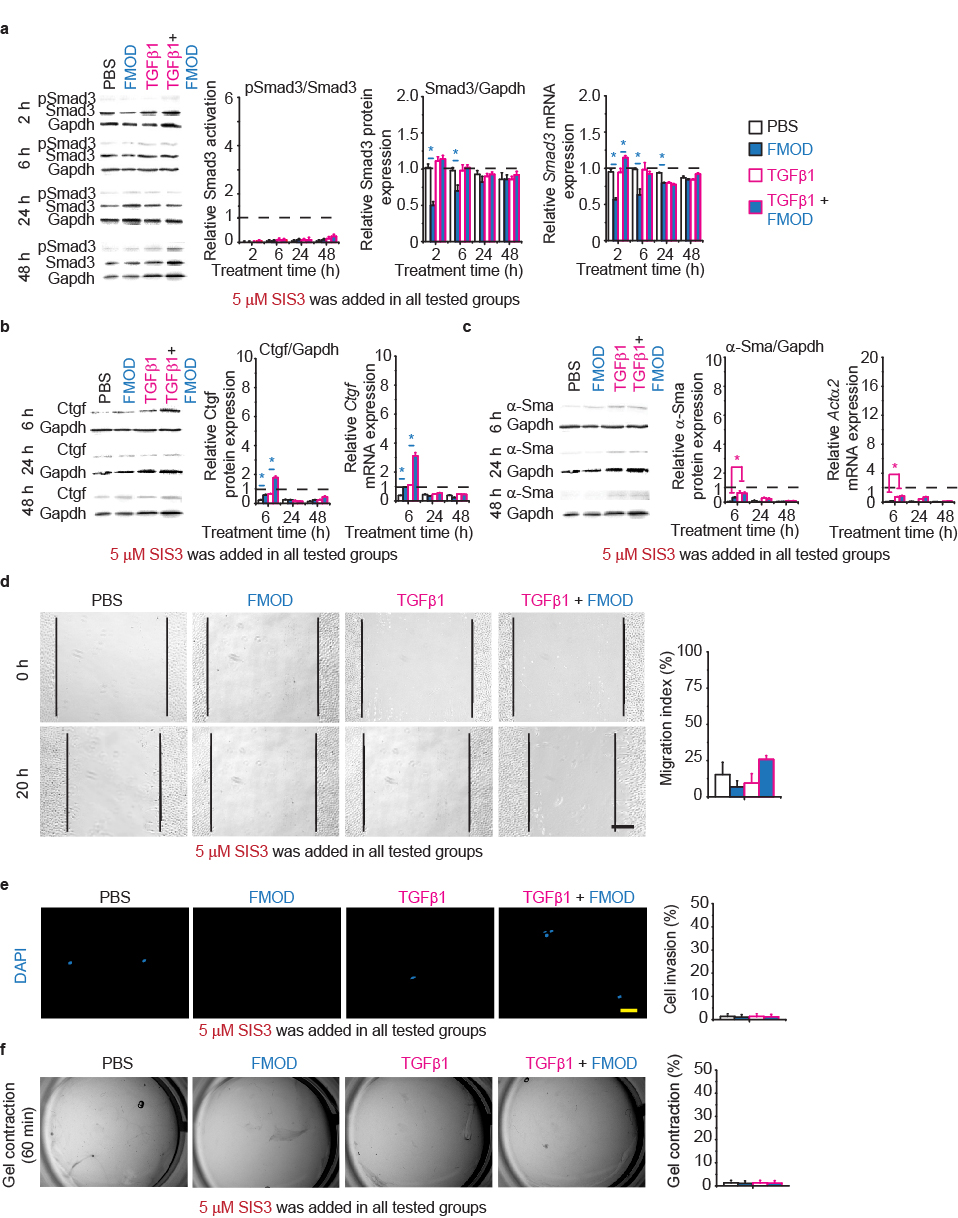

Supplement: Supplementary Figure 10 [file sigtrans201750-s11.jpg]
